# Balloon Pulmonary Angioplasty: A Treatment Option for Inoperable Patients with Chronic Thromboembolic Pulmonary Hypertension

**DOI:** 10.3389/fcvm.2015.00004

**Published:** 2015-02-17

**Authors:** Aiko Ogawa, Hiromi Matsubara

**Affiliations:** ^1^Department of Clinical Science, National Hospital Organization Okayama Medical Center, Okayama, Japan

**Keywords:** thrombosis, pulmonary hypertension, angioplasty, pathology, lung injury

## Abstract

In chronic thromboembolic pulmonary hypertension (CTEPH), stenoses or obstructions of the pulmonary arteries due to organized thrombi can cause an elevation in pulmonary artery resistance, which in turn can result in pulmonary hypertension. CTEPH can be cured surgically by pulmonary endarterectomy (PEA); however, patients deemed unsuitable for PEA due to lesion, advanced age, or comorbidities have a poor prognosis and limited treatment options. Recently, advances have been made in balloon pulmonary angioplasty for these patients, and this review highlights this recent progress.

## Introduction

**Figure 1:**
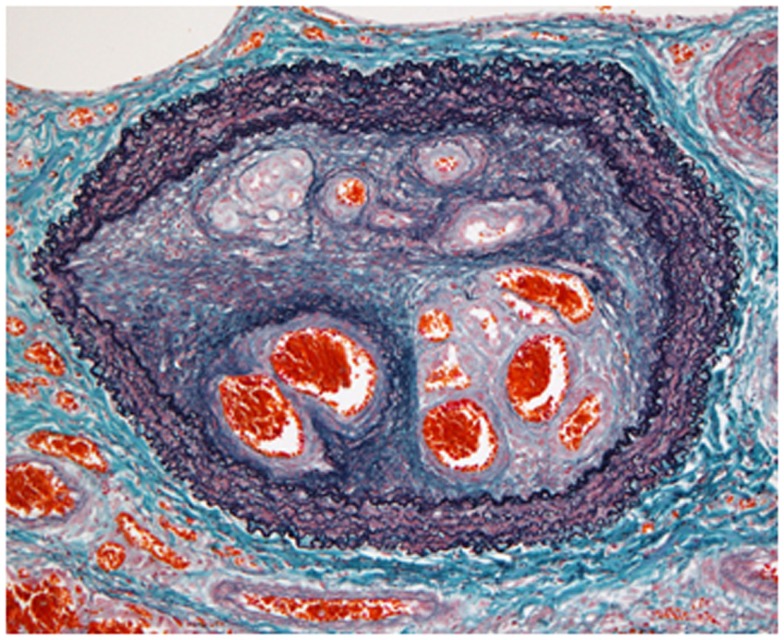
**Organized thrombi in chronic thromboembolic pulmonary hypertension**. The histopathological findings indicated luminal narrowing due to thrombi organized by fibrous intimal hyperplasia, containing characteristic recanalized channels (known as colander lesions) of the pulmonary artery (elastic tissue stain).

Chronic thromboembolic pulmonary hypertension (CTEPH) is a form of pulmonary hypertension classified as Group 4 (1). In CTEPH, stenoses or obstructions of the pulmonary arteries due to organized thrombi can cause an elevation in the pulmonary artery resistance, which may in turn result in pulmonary hypertension (Figure 1). Previously, the prognosis of patients with CTEPH whose mean pulmonary arterial pressure (PAP) is >30 mmHg was very poor, if left untreated, at only 10% at 3 years (2).

The only established and potentially curative treatment currently available for CTEPH is pulmonary endarterectomy (PEA) (3); however, PEA can only be performed at a limited number of institutions at the present time, as the surgical technique requires proficiency and intermittent total circulatory arrest under deep hypothermia (4, 5). Although the postoperative outcome was reportedly worse in patients with distal thrombi, at expert centers, the outcomes of both proximal and distal cases are recently reported to be excellent (5, 6). However, because patients of an advanced age, with comorbidities, and with a poor general condition are ineligible for PEA, not all patients can undergo the surgery. Based on the data from an international registry, 63% of the patients with CTEPH were considered operable, 36% inoperable, and 57% actually underwent surgery (7).

Although patients who are unsuitable for PEA are treated with pulmonary hypertension-specific drugs, the efficacy of these drugs for lowering the mean PAP in patients with CTEPH had not been established (8). Riociguat, a stimulator of soluble guanylate cyclase, has recently been reported to be effective for inoperable CTEPH or persistent/recurrent pulmonary hypertension after PEA (9). The riociguat group was observed to have a significant improvement in the primary endpoint of 6-min walk distance. Pulmonary vascular resistance significantly decreased and the NT-proBNP level and WHO functional class were significantly improved. It is now approved by the U.S. Food and Drug Administration, the European Medicines Agency, and in Japan. However, its long-term efficacy is not yet established. Moreover, medical therapy in CTEPH should not be considered as a replacement for PEA (3) and riociguat is approved only for selected patients as described above.

There is another treatment option for inoperable patients with CTEPH, balloon pulmonary angioplasty (BPA). The latest guideline for CTEPH states that there are numerous concerns and unanswered questions about this technique and its role in CTEPH remains uncertain and requires further evaluation before it can be recommended as an established treatment for CTEPH (3). Recently, advances have been made in BPA and this review highlights its recent progress.

## History of Refined Balloon Pulmonary Angioplasty

**Figure 2:**
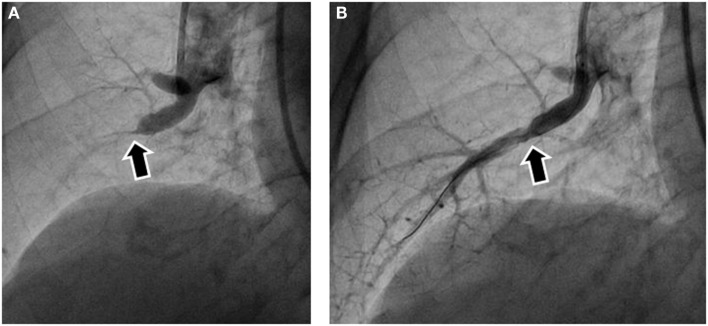
**Pulmonary angiography before and after balloon pulmonary angioplasty (BPA)**. **(A)** Subtotal obstruction was noted in the pulmonary angiography before BPA (arrow). **(B)** Pulmonary angiography after BPA showed blood flow to the peripheral arteries after balloon dilatation. The arrow indicates the same site as the arrow in **(A)**.

Balloon pulmonary angioplasty is an interventional treatment that uses a balloon catheter to dilate pulmonary stenoses (Figure 2). BPA was first developed in the field of pediatric cardiology for treating congenital hypoplastic and stenotic pulmonary arteries (10), and since 1988, BPA has been performed for CTEPH cases that are ineligible for PEA (11). A study published in 2001 summarizing the outcomes for 18 CTEPH cases suggested that BPA was effective (12). However, the procedure required improvement, as the treatment effects were less than those obtained by PEA. Further, BPA was found to be frequently associated with pulmonary edema, which can be fatal. Later, two more cases with inoperable CTEPH were reported to be improved by BPA (13). However, more than 20 years after the first report of BPA for CTEPH, BPA is still not widely accepted as a therapeutic option for inoperable patients.

Since 2004, we have been attempting to improve the BPA procedure at our hospital. Because we had seen many patients with CTEPH who were diagnosed as inoperable and suffered from increasing disability in spite of treatment with pulmonary hypertension-specific drugs. In Japan, there had been no specific drug available for treating CTEPH until September 2014 when riociguat was approved. Considering the high mortality of these patients when untreated, we needed an alternative therapeutic option. We had performed BPA on 68 patients by 2011 and reported efficacy of BPA (14). With only one perioperative death, the safety of BPA was also improved compared with that previously reported (12). With the publishing of BPA studies from other Japanese institutions (15, 16), BPA was covered at the Fifth World Symposium on Pulmonary Hypertension (3). Now, attempts are being made outside of Japan as well, and the efficacy of BPA has been confirmed in selected patients (17–19).

## Indications of BPA

### Patient factors influencing the indication for BPA

Since PEA is the gold standard therapy for CTEPH, patients who are not eligible for PEA are considered as candidates for BPA. Furthermore, patients with residual or recurrent pulmonary hypertension after PEA are also candidates in case repeated surgery is judged to be difficult. Most importantly, the patients should be informed about and understand the risks and benefits of both PEA and BPA before undergoing either treatment.

Contraindications of BPA include iodine allergy, as the use of a contrast medium is essential in BPA. Additionally, in cases with renal dysfunction, the benefits of performing BPA must be weighed against the risks. Severity of pulmonary hypertension may not necessarily be a contraindication of BPA. Although previous reports have indicated a higher mean PAP at baseline is associated with more frequent complications, the patient prognosis will be worse without effective treatment in cases with severe hemodynamics. BPA can be expected to have more powerful effect in these patients. Indeed, recent studies, including mainly severely ill patients with high mean PAP and low cardiac index, reported successful treatment outcomes with BPA (14–16, 20, 21). Age is also often considered, and the safety and efficacy of BPA in elderly patients have already been reported in recent studies (14–16), with the hemodynamic improvements being comparable between younger and elderly patients (22). The prevalence of complications was also comparable between these two groups.

According to the Japanese Circulation Society’s statement regarding BPA, the candidates for BPA are described as follows: (1) unsuitable cases for PEA (surgically inaccessible lesions, surgically accessible but inoperable because of comorbidities, and cases of residual or recurrent pulmonary hypertension after PEA); (2) cases in which conventional therapy is insufficient (WHO functional class ≥III after conventional therapy, mean PAP ≥30 mmHg, or PVR ≥300 dyne ⋅ s ⋅ cm^−5^); (3) patients who provide informed consent (patients who want to be treated with BPA after fully understanding the risks and benefits of both BPA and PEA); and (4) cases without serious complications, multiorgan failure, or iodine allergy (23).

### Indication for each lesion

The most distinct difference between PEA and BPA is the different perception of the indication for lesions. In PEA, the organized thrombi are considered as a whole, and the indication for surgery is made depending on the extent and location of the organized thrombi. In contrast, in BPA, each individual artery is evaluated for indication of BPA based on the lesion type and distribution/location of the lesion.

In patients with CTEPH, lesions are generally present in most arteries, and there is usually a mixture of various lesion types. The classification of CTEPH is currently based on the endarterectomized tissue removed by PEA (24). This is useful as it is easy to understand the distribution of the organized thrombi. However, in performing BPA, the angiographical classification is more useful in evaluating the lesion types of each target lesion for BPA, including pouching defects, webs or bands, intimal irregularities, abrupt vascular narrowing, and complete vascular obstruction (25).

When large amounts of organized thrombi are found in the proximal portion of the pulmonary artery, BPA is not recommended, as the thrombi cannot be removed using this method and surgically easily accessible. Moreover, complete obstructions at the orifices of the segmental arteries that do not show traces of distal arteries or pouching defects are also unsuitable for BPA, as it is extremely difficult for the guidewire to pass through these arteries, although BPA for proximal thrombi has been attempted (26).

Regarding the lesion location, most studies have reported on the treatment of lesions in the segmental or subsegmental arteries in peripheral-type CTEPH patients. In PEA, the more distal the lesion is located, the more difficult the surgery is, and this is why BPA is mainly performed in the distal pulmonary arteries. However, when the lesions are located too distally in the subsegmental pulmonary arteries for the balloon catheter to pass the lesion, BPA may also be unsuitable.

The success rate for each lesion varies among the lesion types. In cases of ring-like stenosis and web and abrupt narrowing, a success rate of almost 100% is achieved, while in cases of the subtotal obstruction type, the success rate is reported to be approximately 90% (23). Conversely, in pouching defects, the success rate is <50%.

## The Procedure of BPA

### The general procedure of BPA

The BPA procedure is approached either through the right internal jugular vein or the right femoral vein, with the internal jugular vein route being better for manipulating the guiding catheter into either the left or right pulmonary artery (12, 14). Using this approach, two operators are needed: one to manipulate the guiding catheter and one to manipulate the guidewire. On the other hand, the femoral vein route has the advantage of one operator being able to manipulate both the guiding catheter and the guidewire; however, manipulating the guiding catheter via the right pulmonary arteries is extremely difficult. A 9-French (Fr) sheath is inserted into the vein, through which a 6-Fr long introducer sheath is advanced into the pulmonary artery. After the sheaths are inserted, heparin is administered to reach an activated clotting time of around 200 s, and an additional 500–1,000 units of heparin are administered every hour. Subsequently, a 6-Fr guiding catheter (multipurpose type or the Amplatz type) is advanced into the pulmonary artery being treated. After performing selective pulmonary angiography, a 0.014″ guidewire is used to cross the lesion. When the treatment is not performed in the upper lobe of the lung, the pulmonary artery must be stretched as much as possible via deep inhalation from the patient, as this facilitates the passage of the guidewire. When the wire has successfully crossed the lesion, a balloon catheter of an appropriate diameter (1.5–10 mm) is selected to dilate the lesion.

Initially, since it was believed that reperfusion itself was the cause of BPA-related lung injury, the target area for one treatment session was limited to two segments on the same side until the mean PAP fell below 30 mmHg (14). There is also an attempt to reduce the rate of lung injury by restricting the treatment area (27). However, the targets and areas for treatment need to be decided according to the operator’s experience and the patient’s lesion type and distribution/location. For example, arteries in the middle or lingular lobes or lesions in the distal to subsegmental arteries are particularly difficult to treat. Because the effect of treating each lesion depends on the size of perfusion area of the segmental arteries containing the lesions, there is an expectation that it is more effective to prioritize treatment of the lesions in the branches of the inferior lobe. Currently, at our institution, there is no limitation regarding the number of lobes targeted in one session but the maximum time of radiographic fluoroscopy in a single session is limited to 60 min. As a consequence, lesions from 4–10 sites are generally treated in a single session. To enhance the therapeutic effect of BPA, it is important to make the area of reperfusion large, which requires repeated treatment with three to four sessions per patient depending on the treatment goal (14–16).

### How to evaluate each lesion

It is critical to evaluate each lesion type and vessel diameter in order to determine the appropriate balloon size for performing angioplasty in BPA. There are several different modalities that have been recently reported as useful, with some of these modalities providing us with clearer images of the lesion. However, we need to consider the feasibility of the modality to be performed in all patients and during all sessions of BPA, as well as the cost and time involved in performing each imaging modality.

Pulmonary angiography is considered the conventional and standard method. To evaluate the lesions in more detail, selective pulmonary angiography should be performed by injecting contrast medium from a catheter inserted into the segmental artery rather than the main pulmonary artery. Intravascular web and band lesions in subsegmental pulmonary arteries are often invisible, and subtotal occlusion with a faint trail of contrast medium in the peripheral arteries can be easily overlooked by ordinary angiography. Accordingly, selective pulmonary angiography should be routinely performed (Figure 2A).

Importantly, angiography does not provide information such as the actual vessel diameter of the lesion or the amount of organized thrombi occupying the lesion. Therefore, intravascular ultrasound (IVUS) is used to evaluate the presence of organized thrombi in the pulmonary arteries and to determine the diameter of the target; subsequently, the balloon is selected depending on the blood vessel diameter as measured by the IVUS (14).

There are some experimental methods to evaluate lesions that are not commonly used. Computed tomography (CT) and/or contrast-enhanced CT have been reported to detect characteristic lesions in CTEPH (28, 29). In one study, cone beam CT was compared with contrast-enhanced CT pulmonary angiography and was found to be useful for the treatment planning of BPA performed distal to the segmental arteries (30). However, not all institutions can perform cone beam CT, and its efficacy has not been compared to that of selective pulmonary angiography. Moreover, the usefulness of optical coherence tomography (OCT) has been reported. The resolution of OCT is high, and OCT allows precise determination of the locations of the target lesions of the pulmonary arteries. It reportedly facilitates the choice of the appropriate balloon size and length (31). Three-dimensional-OCT imaging has been reported to be even more useful in evaluating the lesions (32, 33); however, a downside of this technique is that an image cannot be obtained without having the blood components temporarily eliminated by a jet injection of contrast medium. It sometimes leads to volume overload in severely ill patients with right heart failure. Further, in cases of highly stenotic lesions, the OCT catheter wedges at the lesion and cannot obtain information about lesion beyond the stenosis.

## Efficacy of BPA

In 2001, Feinstein et al. reported that, with an average of 2.7 BPA procedures, the mean PAP of their patient group decreased by 9 mmHg (12). Similarly, three studies reported from Japan in 2012 demonstrated the effectiveness of BPA, with a mean PAP reduction of 14–21 mmHg from baseline (mean PAP, approximately 45 mmHg) with three to four BPA procedures (14–16); refined BPA demonstrated a larger effect in reducing the mean PAP. We reported to achieve treatment outcomes equivalent to those for PEA, observing significant improvements in the WHO functional class, cardiac index, and pulmonary vascular resistance besides mean PAP (14).

Two studies based on smaller case series focusing on other effects of BPA reported on the right ventricular function measured by echocardiography or cardiovascular magnetic resonance imaging (34, 35); both reports concluded that the right ventricular volume index was improved after BPA. Further, ventilatory inefficiency have also been shown to improve after BPA (18, 36).

In our previous report, at the follow-up catheter examination, significant improvements in the hemodynamics and exercise capacity were maintained (14). Since there have been no reported cases of restenosis after BPA to date, its effectiveness seems to be maintained in the long-term, although only balloon dilatation without stenting is performed. Larger and longer observations and analyses are needed. Moreover, currently, there are insufficient data regarding the long-term survival of patients undergoing BPA. One report stated that the 2-year survival rate after BPA treatment was 100% (15), and in another report, 85% patients were alive after 51 ± 30 months of follow-up (18). Considering the high mean PAP at baseline, these data may offer hope for better survival due to BPA.

## Mechanism of Improvement of Hemodynamics by BPA

**Figure 3:**
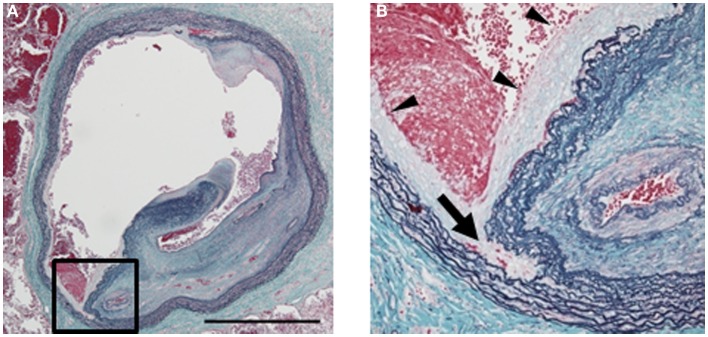
**Histology of a pulmonary artery treated by balloon pulmonary angioplasty (BPA) [cited from Ref. (38)]**. **(A)** A large lumen was formed due to dissection in the media of a pulmonary artery by BPA (elastic tissue stain, low magnification; bar: 1 mm). **(B)** High magnification of the dissection site [square in **(A)**; elastic tissue stain]. Dissection occurred in the middle of the media (arrow). Newly formed intima is observed on the inner surface (arrowheads). One of the recanalized lumina in the organized thrombi is also seen on the right.

Pulmonary endarterectomy, the gold standard procedure for treatment of CTEPH, involves removal of intraluminal thrombi from inside the pulmonary arteries; on the other hand, BPA does not remove thrombi from the pulmonary arteries. Therefore, we questioned why and how BPA can lead to improving the hemodynamics without removal of the thrombi. Moreover, we questioned why, unlike for the coronary arteries, restenosis does not occur despite the fact that ballooning alone is performed without using any stents. Previously, we pathologically examined lesions after BPA and found that the only change in the organized thrombi was a small incision (37). A subsequent investigation of an autopsy case revealed that BPA had caused dissection of the tunica media in the treated sites, and that the organized thrombi had partially detached from the vascular wall (Figure 3) (38). Thus, this procedure involves the same technique as PEA, only differing in the fact that the thrombi are not extracted from inside the blood vessel, but rather forced to one side to make the lumen larger. Following BPA, parts of the vascular wall exhibited thinning due to dissection; these thin areas of the vascular wall are then subjected to PAP, leading to expansion of the lumen diameter over time without causing restenosis.

## Complications of BPA

### Complication rate of BPA

In the first report of BPA, the in-hospital death rate was 5.6% (12), whereas it was slightly lower, at 0–3.4%, in the following reports (14–16). Issues associated with BPA include overcoming lung injury following the procedure. Lung injury was previously thought to be caused by reperfusion edema (12), which is also an important postoperative complication of PEA. In severe cases, mechanical ventilation and percutaneous cardiopulmonary support are required, and the lung injury may be fatal. Although BPA could be considered minimally invasive, BPA-related lung injury reportedly occurs in 9.6–60% of the cases (14, 16, 18). This frequency is higher than in PEA, suggesting that the mechanism causing lung injury in BPA may differ from that in PEA. Cytokine release or a sudden increase in blood flow to the peripheral arteries has been suspected to be the cause of lung injury after PEA (39), and these mechanisms may also be involved in inducing lung injury in BPA.

However, in the case of BPA, vascular injury caused by the guidewire or guiding catheter, or injecting contrast medium with high pressure may also play a role in inducing lung injury. Indeed, by reviewing CT images after BPA and pulmonary angiography, consolidation was observed in the area where BPA was performed in a previous study, and this could also explain why there may be a learning curve for reducing BPA-related lung injury (14). The complication rate for each lesion varies among the lesion types, similar to the fact that the success rate varies depending on the lesion types. In cases of ring-like stenosis and web and abrupt narrowing, a complication rate is 2%. The complication rate is approximately 16% in cases of the subtotal obstruction type, and 10% in pouching defects (23).

In addition, after establishing that BPA is actually dissecting arterial wall (38), some parts of BPA-related lung injury have been found to occur at the dilation site, resulting in overdilatation or progression of dissection of the pulmonary arteries. In addition, even if no signs of complications are observed immediately after BPA, expansion of the thin vessel walls caused by the detachment of organized thrombi may progress in cases with extremely high PAPs, which in turn can lead to oozing ruptures, as observed in aortic dissection or evident pulmonary hemorrhages. There is also a risk of pulmonary artery perforation or vessel rupture with a guide wire (14). It may require emergent transcatheter coil embolization or the use of covered stents (40).

### How to reduce the risk of lung injury

Since lung injury was initially thought to be caused by the same mechanism as that occurring after PEA, we attempted administering epoprostenol to lower the PAP and using methylprednisolone and non-invasive positive-pressure ventilation to prevent pulmonary edema (14). However, our findings suggested that these measures could not prevent BPA-related lung injury. Later, an attempt to predict the risk of pulmonary edema resulting from BPA was made by using pulmonary angiography (27). However, if one of the major causes for lung injury is catheter-related, as described above, it might be possible to reduce the risk purely by refining the technique used. For example, using a guidewire with a tip load as small as possible, stopping the tip of the guidewire within the angiographically visible range of the distal vessels, and injecting the contrast agent gently, may all help reduce the risk of lung injury. However, these measures alone are likely not enough, as the complication after BPA has not been eliminated. Further, when lesions have a large amount of organized thrombi, balloon dilation itself may cause excessive extension of the pulmonary vascular wall, likely resulting in vascular injury. Maximum dilatation is considered to be related to more extensive injuries to the vascular wall of the lesion. In order to reduce the risk of vascular wall injuries, the dilatation of the lesion site has to be kept to a minimum. Feinstein et al. reported that high PAP prior to BPA led to a high frequency of postoperative lung injury after BPA (12). Thus, BPA must be performed with particular caution in severe cases.

When keeping the dilatation of the lesion site small, the effectiveness of BPA decreases. The therapeutic effect of BPA is directly proportional to the number of vessels treated (14); thus, even if the dilatation of the lesion sites is kept small, the therapeutic effect of one session can be maintained by treating several lesions in one session. However, if the operator has insufficient experience, treating many lesions at one time leads to an increased risk of vascular injury at many lesions. Thus, the most realistic countermeasure seems to be increasing the number of BPA repetitions.

## Present Limitations of BPA and Future Directions

There is a need for further understanding and information regarding BPA. A novel classification of BPA suitability according to the lesion type is needed to better select patients who would be most benefited from BPA. Improved strategies to overcome the complications associated with BPA must be established. BPA-specific improvements to devices, such as guiding catheters, guidewires, and balloon catheters, are required. Randomized control trials to evaluate the superiority of BPA over drug therapy and studies on the cost-effectiveness of BPA procedures are needed. Furthermore, long-term data on the risks of restenosis are lacking, as are data on the need for stenting in these patients. Long-term survival and efficacy need to be clarified.

There are also demands for BPA performed in adult patients with isolated peripheral pulmonary artery stenosis (41), and in such cases, there might be a need for stenting (42). We also do not know how to treat very distal arteries with a diameter <100 μm, which can currently not be treated either by PEA or BPA (37).

## Conclusion

Balloon pulmonary angioplasty is a novel treatment that can potentially provide marked improvements in subjective symptoms and hemodynamics in CTEPH patients ineligible for PEA. However, further refinements of the strategy to reduce complications, improvements in the simplicity of the treatment, and evaluation of the long-term follow-up results are needed before BPA can be recommended as an established treatment for CTEPH.

## Author Contributions

AO performed the conception of the work, the acquisition, and interpretation of the data for the work and manuscript writing. HM performed the conception of the work and manuscript writing.

## Conflict of Interest Statement

Aiko Ogawa has received lecture fees from GlaxoSmithKline K.K., Actelion Pharmaceuticals Japan Ltd., and Nippon Shinyaku Co., Ltd. and a material for research from Boehringer Ingelheim International GmbH. Hiromi Matsubara has received lecture fees from GlaxoSmithKline K.K., Actelion Pharmaceuticals Japan Ltd., and Pfizer Japan Inc. and research grants from GlaxoSmithKline K.K.
